# Health concerns and government distrust: Variation in types of COVID-19 vaccine hesitancy by racial and ethnic group before and at universal vaccine eligibility in the US

**DOI:** 10.1371/journal.pgph.0004942

**Published:** 2026-06-03

**Authors:** Meg Ellingwood, Alex Reinhart, D. Phuong Do, Robin Mejia

**Affiliations:** 1 Department of Statistics & Data Science, Carnegie Mellon University, Pittsburgh, Pennsylvania, United States of America; 2 Zilber College of Public Health, University of Wisconsin–Milwaukee, Milwaukee, Wisconsin‌‌, United States of America; 3 Department of Biostatistics, University of Washington, Seattle, Washington‌‌, United States of America; PLOS: Public Library of Science, UNITED STATES OF AMERICA

## Abstract

The development and deployment of COVID-19 vaccines were crucial to combating the global pandemic, but vaccine hesitancy posed a challenge and remains a crucial problem today. Understanding who delays or refuses vaccination and why can help with current and future vaccine campaigns by suggesting both target audiences and messaging. Because hesitant individuals report different reasons for their hesitancy, distinguishing between different types of hesitancy can help identify who will benefit from which strategies. Using 311,494 responses from the U.S. COVID-19 Trends & Impact Survey (CTIS), we used Latent Class Analysis to examine distributions of concurrently reported reasons for hesitancy, comparing distributions of responses across racial and ethnic groups. Using responses from both the early phase of vaccine availability and after all U.S. adults became eligible in the spring of 2021, we investigated differences between prospective vs. lived hesitancy. LCA revealed three different types of COVID-19 vaccine hesitancy. Some respondents primarily reported health-related concerns as reasons for hesitancy, while others indicates distrust of the government and vaccines, but the majority report fewer distinct reasons. In February 2021, White, Black, and Hispanic respondents had similar distributions of these types, but by May the results diverged. In May, White respondents were more likely to report trust concerns than Black or Hispanic respondents, who were more likely to report health concerns as their reasons for continued hesitancy. Our results contribute to the development of a more nuanced picture of COVID-19 vaccine hesitancy and the motivations behind it. The distinction between *health-concerned* and *distrustful* types of vaccine hesitancy highlights the importance of confidence in vaccine uptake, and in targeted strategies to address address hesitancy.

## Introduction

The development and delivery of effective COVID-19 vaccines was a milestone public health achievement, saving lives and enabling a return to normal life. In December 2020, nearly a year after the first COVID-19 case was diagnosed, the first two COVID-19 vaccines received emergency use authorization in the United States. The Centers for Disease Control and Prevention (CDC) offered guidelines for phased distribution of the initially limited supply, and states implemented a range of distribution policies. Broadly, healthcare workers and residents of long-term care facilities were the first to be eligible for the vaccine, followed by everyone over the age of 75. Access expanded over the following weeks, with specifics decided by each state. Finally, by May 2021, all adults were eligible [1,2].

Vaccine hesitancy, already concern in public health, was anticipated to be a problem with the COVID-19 vaccine [3]. Traditional frameworks for vaccine hesitancy highlight the importance of convenience, confidence, and complacency, known as the “3Cs,” as factors behind vaccine hesitancy or demand [4].

In 2015, the Sage Working Group on Vaccine Hesitancy developed a definition including these factors but highlighting the importance of context: *Vaccine hesitancy refers to delay in acceptance or refusal of vaccination despite availability of vaccination services. Vaccine hesitancy is complex and context specific, varying across time, place and vaccines. It is influenced by factors such as complacency, convenience and confidence [4].*

The working group developed a vaccine hesitancy determinants matrix, with 21 determinants of hesitancy grouped under three overarching categories: contextual influences (including communications/media, leadership/lobbying, historical influences, religion/culture/demographics, politics/policy, geography, and perception of the pharmaceutical industry), individual and group influences (personal/family/community experience, health beliefs/attitudes, knowledge, trust in health systems/providers, perceived risk/benefit, social norms, and vaccine/vaccination specific issues (scientific evidence of risk/benefit, newness of a vaccine or formulation, mode of administration, vaccination program, reliability of supply, vaccination schedule, costs, recommendations of health care professionals‌‌. (See [4] for full text of the determinants.)

Others expanded the 3Cs model directly to include calculation (searching for information) and collective responsibility, forming the 5C’s framework [5]. These constructs have been studied theoretically and in practice across a range of contexts; for example, pre-pandemic studies found they explained 20–40% of the variance in vaccination behavior in Germany [5]. In the U.S., these factors vary across groups and may reflect a history of racial and socioeconomic disparities in vaccination. Before the pandemic, racial and ethnic disparities in childhood vaccination rates were improving [6]; however, Black and Hispanic children from lower socioeconomic backgrounds remained less likely to receive the MMR (measles-mumps-rubella) vaccine. Similarly, Black Americans were less likely than white Americans to receive the annual flu vaccine [7].

However, the COVID-19 pandemic created new context [8,9]. The extreme salience of the disease was predicted to motivate uptake [10]. However, some Americans showed concerns about the development and approval process [11]. Additionally, because the vaccine was authorized in December 2020 and many people did not have access until late spring 2021, initial studies assessed how people believed they would respond when a vaccine became available, rather than how they actually responded, often much later. These studies implicitly created a new definition for vaccine hesitancy that includes intention to delay acceptance or refuse a not-yet-available vaccination when it becomes available.

Previous work both before and during the vaccine rollout identified many individual factors associated with hesitancy toward the COVID-19 vaccine, including gender, age, income, education, and occupation [11–20].

Hesitant individuals reported many reasons, from worries about safety and side effects to distrust of the government and the development process for the new vaccines [12,18].

Race and ethnicity were also associated with hesitancy in the U.S. [12,19], with vaccine hesitancy higher among many non-White groups early on, with the exception of Asians. This was particularly concerning given the disproportionate impact of the COVID-19 pandemic in Black and Hispanic communities.

The reasons given for hesitancy in Spring 2021 also varied among different demographic groups. Overall, fear of side effects was the single most commonly reported reason within each racial/ethnic group, but the next-most common reason differed by race/ethnicity: White respondents were more likely to say they didn’t trust the government compared with wanting to wait and see if the vaccine was safe, while the reverse was true of Hispanic and Black respondents [15].

Numerous reasons for hesitancy are potentially interconnected. However, prior studies commonly analyzed reasons selected for vaccine hesitancy individually, ignoring relationships between reasons. Further, most studies snapshots in time. In this study, informed by the SAGE Vaccine Hesitancy Determinants Matrix, we employ Latent Class Analysis to identify groups of hesitancy reasons based on co-occurance patterns of hesitancy reasons. We then examine how the types of hesitancy vary between different racial/ethnic groups. Finally, we assess patterns across time, comparing hesitancy both and after before universal vaccine availability, which we refer to as prospective hesitancy and lived hesitancy.

## Methods

### Ethics statement‌‌

Survey respondents gave written informed consent to participate in CTIS. This analysis was allowed under the overall survey consent. The Carnegie Mellon University Institutional Review Board approved the study (STUDY2022_00000113).

### Data summary

The US COVID-19 Trends & Impact Survey (CTIS) was a large, daily cross-sectional online survey conducted by the Delphi Group at Carnegie Mellon University in partnership with Meta and the University of Maryland from April 2020 through June 2022 [21]. Participants were recruited from the Facebook monthly adult (age 18+) active user base using stratified random sampling via an invitation above their News Feed. Those who responded were sent to a Qualtrics survey administered by Carnegie Mellon University. Survey respondents gave written informed consent to participate. The survey did not collect any personally identifying information [21,22].

Questions on vaccine hesitancy were introduced on February 8, 2021, and remained unchanged through May 20, 2021. Our analytical samples are composed of respondents from February 8 through February 28, during a period when vaccines were only available to a limited subset of the population (*prospective hesitancy* sample) and from May 1 to May 20, when all adults were eligible for the vaccine (*lived hesitancy* sample). Data for this study were accessed April 18, 2022.

Full question and answer choices used in this analysis are provided in the S1 Appendix. Respondents who selected that they had not yet received one or more doses of a COVID-19 vaccine, and responded that they “probably would not” or “definitely would not” accept a vaccine if one were offered to them that day, were classified as vaccine hesitant. Hesitant respondents received a follow-up question asking them to select one or more reasons for choosing not to be vaccinated from a list of 15 options: concerns about side effects, having an allergic reaction, unsure if the vaccine will work, don’t believe they need it, don’t like vaccines generally, doctor has not recommended, want to wait and see if it is safe, others need it more, worried about cost, don’t trust the government, against religious beliefs, don’t trust COVID-19 vaccines specifically, health condition, pregnant/breastfeeding, and other.

Race and ethnicity of respondents were ascertained through a two-part question that asked about Hispanic or Latino origin and race. Race-stratified analyses were conducted with respondents who identified as non-Hispanic White, non-Hispanic Black, or Hispanic, as those groups had adequate sample size for analysis.

### Latent class analysis

To assess how respondents tended to select multiple hesitancy reasons together, we performed latent class analysis (LCA), a form of mixture model in which individuals are assumed to belong to unobserved groups called latent classes [23]. These unobserved latent classes are modeled based on observed categorical variables. In this study, we applied LCA to assess patterns for COVID-19 vaccine hesitancy by modeling latent classes of hesitancy types, based on reasons selected by survey respondents. Each latent class represents a distinct response pattern, with a vector indicating the probabilities of selecting specific hesitancy reasons. For example, a respondent in Hesitancy Class A has a certain probability of selecting Hesitancy Reason X. This approach enables the estimation of common hesitancy response patterns and the probability that each respondent belongs to each class. We included race/ethnicity as a categorical grouping variable, allowing different latent structures across race/ethnicity [24]. This enabled comparison of the identified classes, and the response probabilities, between racial/ethnic groups.

Mathematical details on the LCA model are provided in S1 Appendix. Goodness-of-fit tests using likelihood ratios and AIC were used to compare possible models [25]. The final number of latent classes selected was based on balancing the results of the likelihood ratio tests and AIC with interpretability of the resulting model.

All analyses were conducted in R (Version 4.1.1, [26]), using the glca package [27].

### Sensitivity analysis

Because incorporating response weights was not feasible for the modeling methods used in this study, all results reported are based on unweighted survey data. As a sensitivity analysis, we compared the distribution of survey weights by class membership probabilities, to assess whether the size of classes would have been affected by inclusion of survey weights. Details on the sensitivity analysis are provided in S1 Appendix

## Results

### Study sample and participant characteristics

**Fig 1:**
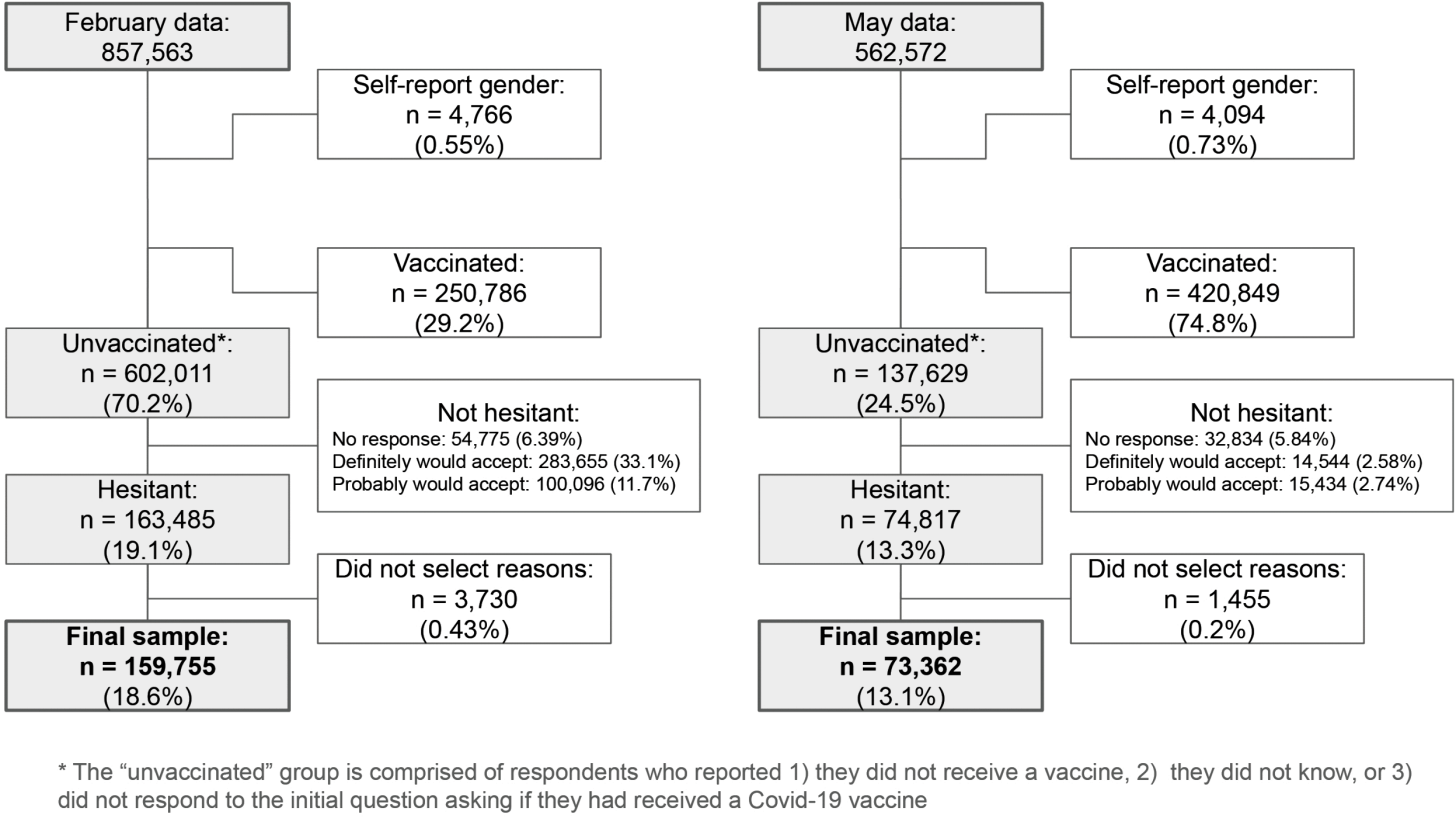
Processing of respondents to isolate “hesitant” sample. Latent class analysis was performed only on participants who responded to the item asking their reasons for hesitancy. Percentages at each stage are out of the original sample size (i.e., all February or all May respondents).

The sample selection is shown in Fig 1. To evaluate prospective and lived hesitancy, we analyzed survey responses from February (N = 857,563) and May 2021 (N = 562,572) separately. Because previous analysis of CTIS data (e.g., [19]) identified high rates of adversarial response patterns among respondents who selected self-described gender (e.g., writing in anti-trans slurs), we excluded participants who selected self-described gender from the analysis (N = 4,766, 0.55%, in February; N = 4,094, 0.73%, in May). We then identified respondents who were unvaccinated. From those, we selected those who indicated they would “probably not” or “definitely not” accept a vaccine if they were offered it on that day (N = 236,677 in February and N = 74,817 in May). We then excluded participants who did not respond to the question asking the reasons for their hesitancy, yielding an analytical study sample of 159,755 individuals in February, and 73,362 individuals in May.

The demographic makeup of the February and May samples is presented in Table 1. Demographic patterns were broadly similar across the two time periods. The sampling process permitted respondents from February to be sampled in May, and privacy considerations in the survey design prevented tracking of respondents over time; however, any overlap in the samples is expected to be small.

**Table 1:** Demographics of final sample of hesitant participants who reported their hesitancy reasons.

	February	May	Overall
	(N = 159755)	(N = 73362)	(N = 233117)
**Gender**			
Male	43286 (27.1%)	23400 (31.9%)	66686 (28.6%)
Female	86125 (53.9%)	34731 (47.3%)	120856 (51.8%)
Non-binary	1004 (0.6%)	595 (0.8%)	1599 (0.7%)
Prefer not to answer	3905 (2.4%)	2586 (3.5%)	6491 (2.8%)
Missing	25435 (15.9%)	12050 (16.4%)	37485 (16.1%)
**Age (Years)**			
18-24	9411 (5.9%)	3377 (4.6%)	12788 (5.5%)
25-34	23685 (14.8%)	9912 (13.5%)	33597 (14.4%)
35-44	26273 (16.4%)	12082 (16.5%)	38355 (16.5%)
45-54	26719 (16.7%)	12837 (17.5%)	39556 (17.0%)
55-64	26084 (16.3%)	12478 (17.0%)	38562 (16.5%)
65-74	15672 (9.8%)	7494 (10.2%)	23166 (9.9%)
75+	5452 (3.4%)	2673 (3.6%)	8125 (3.5%)
Missing	26459 (16.6%)	12509 (17.1%)	38968 (16.7%)
**Education**			
Less than high school	7085 (4.4%)	2677 (3.6%)	9762 (4.2%)
High school graduate / GED	33041 (20.7%)	13531 (18.4%)	46572 (20.0%)
Some college	39546 (24.8%)	17970 (24.5%)	57516 (24.7%)
2 year degree	17418 (10.9%)	8435 (11.5%)	25853 (11.1%)
4 year degree	21450 (13.4%)	10776 (14.7%)	32226 (13.8%)
Professional degree (e.g., MD, JD, DVM)	2346 (1.5%)	1383 (1.9%)	3729 (1.6%)
Doctorate	1717 (1.1%)	1049 (1.4%)	2766 (1.2%)
Master’s Degree	8779 (5.5%)	4256 (5.8%)	13035 (5.6%)
Missing	28373 (17.8%)	13285 (18.1%)	41658 (17.9%)
**Race/Ethnicity**			
White	89997 (56.3%)	43774 (59.7%)	133771 (57.4%)
Black	12287 (7.7%)	3062 (4.2%)	15349 (6.6%)
Multiple/Other	8635 (5.4%)	5592 (7.6%)	14227 (6.1%)
Native American	1915 (1.2%)	808 (1.1%)	2723 (1.2%)
Asian	1283 (0.8%)	335 (0.5%)	1618 (0.7%)
Pacific Islander	329 (0.2%)	117 (0.2%)	446 (0.2%)
Hispanic	16630 (10.4%)	6028 (8.2%)	22658 (9.7%)
Missing	28679 (18.0%)	13646 (18.6%)	42325 (18.2%)

### Classes of prospective hesitancy, February 2021

In February, 19.06% of all respondents, and 27.2% of unvaccinated respondents, were identified as hesitant. Because the COVID-19 vaccine was not yet widely available during this time period, we consider this hesitancy to be primarily prospective: people’s belief about what they would do when a not-yet-possible option became available.

We initially fit latent class models with 3–30 classes. As the number of classes increased, AIC consistently decreased. Likelihood ratio tests confirmed improved model fit with more classes, likely because of the large sample size. Because interpretability becomes increasingly difficult as the number of classes increases, we restricted further analysis to models with fewer than 10 classes. Examination of these models revealed that beyond three classes, allowing additional classes primarily subdivided already small groups without providing meaningful distinctions. Thus, for parsimony, we determined that three classes provided the best balance between model fit and interpretability.

#### Among all race/ethnicity groups.

**Fig 2:**
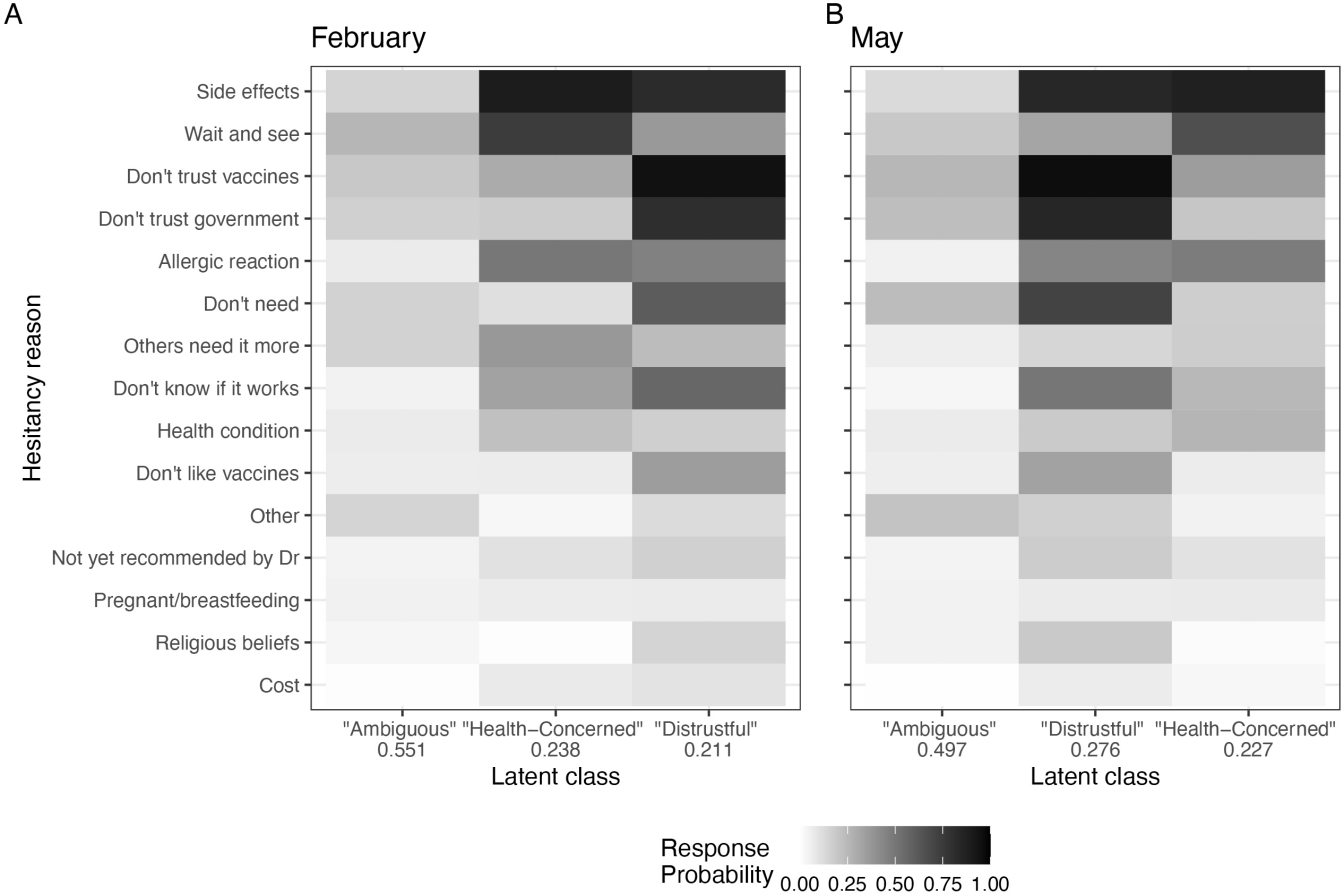
Latent class analysis among all race/ethnicity groups. A: Results of LCA with three classes on full sample of participants in February 2021. Cell shading indicates the probability of selecting each hesitancy reason for members of each class. Numbers on the *x* axis indicate the average membership probability for each class. Hesitancy reasons are arranged by overall descending prevalence (regardless of class) in the February sample. B: Results of LCA with three classes on full sample of participants in May 2021.

The largest class had an average membership probability of 55.1% (Fig 2, panel A; Table A in S1 Appendix provides point estimates and standard errors). On average, respondents in this class selected 1.61 reasons, indicating a tendency to choose one to two specific reasons for hesitancy. No particular reasons stood out for this group. Wanting to “wait and see” if the vaccines were safe had a probability of 25.8% to be selected, but several other reasons had comparable chance of selection, including that they “don’t trust COVID-19 vaccines” (19.5%). In contrast, “don’t like vaccines” in general had a much lower probability of only 6.2%. We labeled this group the *ambiguous* class.

The second class had an average membership probability of 23.8%. Unlike the first class, multiple reasons emerged as notable. The top two reasons were “concern about possible side effects”, with a response probability of 89.8%, and the desire to “wait and see” if the vaccine was safe, with a probability of 74.7%. Members of this class were also more likely than other classes to express concerns about “allergic reactions” and feelings that “others need it more”. Some also indicated they “don’t know” if the vaccine would work. The average number of reasons selected by this class was 4.01. Since the most prominent reasons for this class were related to worries about well-being and the effects of the vaccine on their immediate health, we labeled this group the *health-concerned* class.

The third class was slightly smaller, with an average membership probability of 21.1%. However, it had the highest average number of selected reasons, at 5.96. The most commonly selected reason was that they did not trust the COVID-19 vaccines, with a probability of 94.8%. Respondents in this class also frequently indicated that they were worried about side effects (82.6%) and that they did not trust the government (80.9%). They were more likely than those in other classes to report that they “don’t like vaccines” in general, and also that they “do not need” the vaccine. Because distrust-related reasons were central to this class, we labeled it the *distrustful* class.

#### Across race/ethnicity.

**Fig 3:**
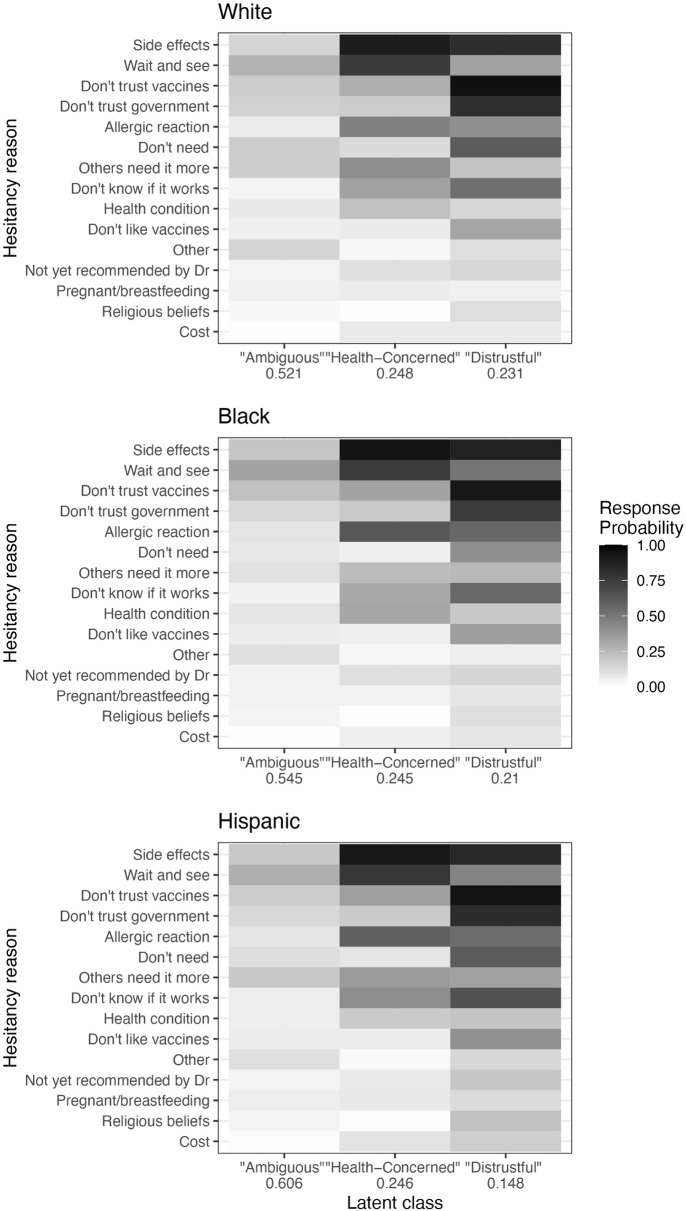
Results of LCA with three classes for White, Black, and Hispanic respondents in February 2021. Cell shading indicates the probability of selecting each hesitancy reason for members of each class. Numbers on the *x* axis indicate the average membership probability for each class. Hesitancy reasons are arranged according to overall prevalence (regardless of class or race/ethnicity) in the February sample.

Three-class LCA models fitted separately for the White, Black, and Hispanic respondents revealed that within each racial/ethnic group, an ambiguous class, a health-concerned class, and a distrustful class consistently emerged. The classes also exhibited similar relative proportions to each other: the ambiguous class was the largest, followed by health-concerned, and then distrustful. Response probabilities and relative class membership probabilities for each group’s model are displayed in Fig 3; probabilities on the scale of 0–1 with standard errors are provided in Table C in S1 Appendix.

However, there were subtle differences in the distribution of reasons within each class across racial/ethnic groups. For example, in the “distrustful” class, Black respondents were 15 percentage points more likely to report concerns about “allergic reactions” than their White counterparts (see Table C in S1 Appendix). They were also 17 percentage points more inclined to indicate that they wanted to “wait and see” if the vaccine was safe. Similarly, Hispanic respondents were more likely to select health-related reasons within the “distrustful” class, compared to “distrustful” White respondents.

### Three types of lived hesitancy, May 2021

In May, 13.3% of all respondents and 54.4% of respondents who were unvaccinated were coded as vaccine hesitant. As the vaccine was available to all US adults at this point, we consider this to be lived vaccine hesitancy. Using the sample that provided reasons for their hesitancy, we again fit models with 3–30 classes and observed that AIC improved with additional classes. Again, the addition of classes resulted in splitting already-small classes, and for parsimony and facilitation of comparison across time, we opt again to proceed with three classes.

#### Among all race/ethnicity groups.

Response probabilities and relative class membership probabilities for May 2021 are displayed in Fig 2, panel B (Table B in S1 Appendix provides point estimates with standard errors). The three-class LCA model fit on the May 2021 sample resulted in similar classes of response patterns found for the February prospective hesitancy sample. Characterized by relatively low probability of selection across many reasons, the ambiguous class was once again the largest. Individuals in this class selected an average of 1.66 reasons, and the probability of class membership was 49.7%, slightly lower than in February. However, by May 2021, the relative prevalence of the two remaining classes had flipped. The second-largest class was the distrustful class, with a membership probability of 27.6%, and the health-concerned class was the smallest, with a membership probability of 22.7%.

#### Across race/ethnicity.

**Fig 4:**
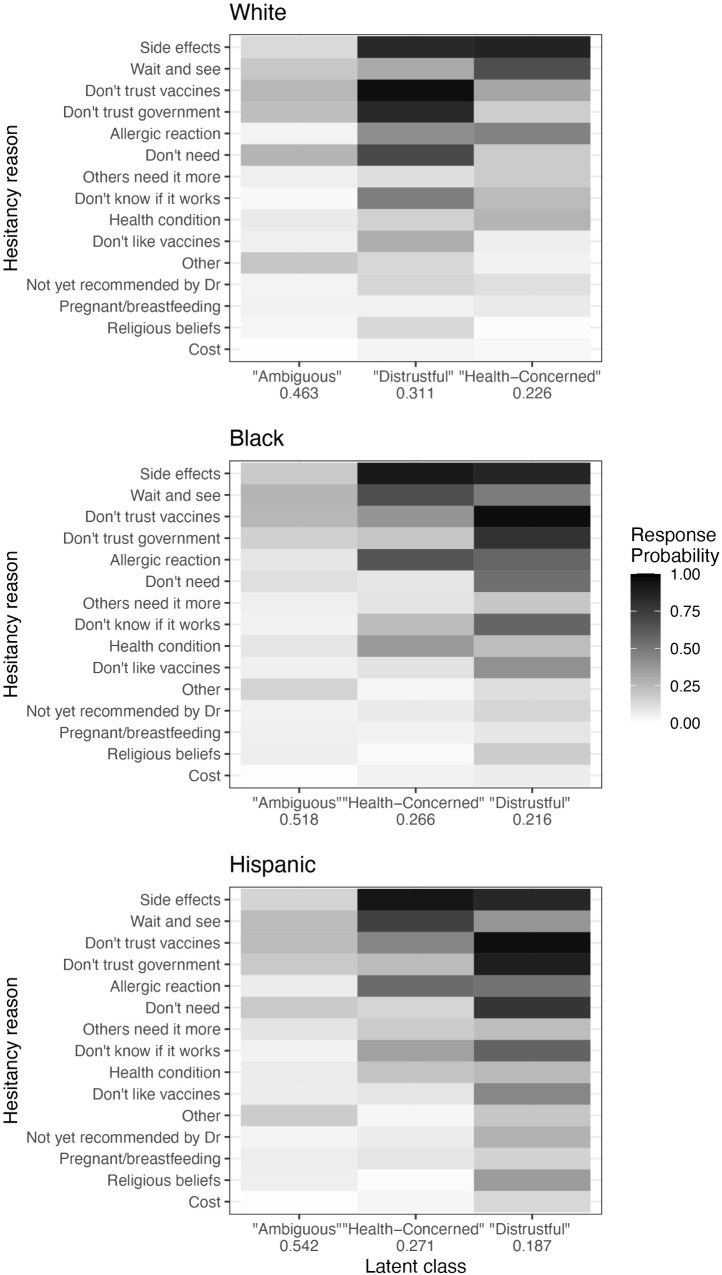
Results of LCA with three classes modeled on White, Black, and Hispanic respondents in May 2021. Cell shading indicates the probability of selecting each hesitancy reason for members of each class. Numbers on the *x* axis indicate the average membership probability for each class. Hesitancy reasons are arranged according to overall prevalence (regardless of class or race/ethnicity) in the February sample, to facilitate comparison to the previous models.

Consistent with the February analysis, we again fit three-class LCA models for the May 2021 White, Black, and Hispanic respondents separately. The response probabilities and class membership probabilities for these models are presented in Fig 4; Table D in S1 Appendix provides point estimates and standard errors.

In May, similar classes again emerged within each race/ethnicity sub-analysis: one ambiguous group characterized by low probabilities of individual selections, one dominated by distrustful reasons, and one focused on health concerns. Within each of the racial/ethnic groups, respondents are most likely to fall into the ambiguous category, but the relative size of this class decreased compared to February.

However, the models for each racial/ethnic group differ in the relative size of the second and third classes. As in the overall model for May 2021, White respondents had a higher probability of falling into the distrustful class (membership probability of 31.1%) and a lower probability for the health-concerned class (membership probability of 22.6%). However, for Black and Hispanic respondents, the health-concerned class remained more common (membership probabilities of 26.6% and 27.1%, respectively), compared to the distrustful class (membership probability of 21.6% for the Black group and 18.7% for the Hispanic group), mirroring the February results.

Also similar to the February analysis, the distrustful class for Black and Hispanic respondents differed from the distrustful class for Whites, again with Black and Hispanic respondents having a higher probability of selecting health-related reasons.

### Sensitivity analysis

As noted above, the LCA model did not incorporate survey weights in the analyses. However, a sensitivity analysis (described in the Methods) found that the distribution of survey weights was similar across classes, indicating that inclusion of the weights would be unlikely to change the relative class size.

## Discussion

Latent class models fit on data from February and May 2021 revealed three distinct classes of COVID-19 vaccine hesitant respondents. Notably, similar classes arose when separate models were fit on the entire hesitant samples for February and May, and again, when separate models were fit on White, Black, and Hispanic subgroups at each of those time points. These results indicate that it is possible to identify consistent sets of belief patterns in all groups that were studied, both early in the vaccine rollout, when most people were not eligible for vaccination and thus reporting prospective hesitancy, and in May 2021, when all US adults were eligible and vaccine availability was widespread. Further, these belief patterns differ from each other in meaningful ways.

The two most interpretable classes identified by the model differ considerably. Health-concerned hesitancy emphasized concerns about side-effects, a desire to wait and see if the vaccine is safe, and concerns about allergies or not knowing if the vaccine works. Distrustful hesitancy was associated with a high probability of not trusting the COVID-19 vaccine, having concerns about side effects, and not trusting the government.

This analysis also highlights the challenges of studying vaccine hesitancy before a vaccine is available. Traditional vaccine hesitancy has been defined in terms of vaccination behaviors, such as “delay in acceptance or refusal of vaccination despite availability of vaccination services,” and factors including convenience, confidence, and complacency have been shown to play a role [4]. In this study, we define hesitancy as stated unwillingness to accept a vaccine were if offered to the person today. Conversely, vaccine acceptance was defined as a stated willingness to receive a vaccine, a definition that included people who are not yet vaccinated among the vaccine accepting, even in May when eligibility was not an issue. This definition is similar to others developed to measure hesitancy to COVID-19 vaccines before they were widely available [8], but the lack of a clear measurement norm during this period highlights the challenge of measuring and understanding hesitancy to a new vaccine in during a public health emergency.

Despite those challenges, our findings suggest that some of the previously studied constructs in vaccine hesitancy also play a role in COVID-19 vaccine hesitancy [5]. For example, the emergence of the health-concerned class supports a need to build confidence in the COVID-19 vaccine.

The emergence of a distrustful class could be viewed similarly, but is more concerning, as it indicates a broader distrust of government institutions. The significance of level of trust is consistent with research linking political environment and political views to COVID-19 vaccine attitudes [28]. Building confidence in government and institutions is therefore a distinct challenge from addressing health-related concerns.

While the classes identified in this study align conceptually with the 3C and 5C models of vaccine hesitancy, they do not map directly onto those models’ constructs. Thus, this work supports both the importance of addressing root causes of vaccine hesitancy and advancing theoretical understanding while also reinforcing the need to assess hesitancy within specific contexts, following the recommendations of the SAGE Working Group on Vaccine Hesitancy and other recent studies. For example, studies of healthcare providers in Brazil found that the 5C framework did not apply to that population [29]. Similarly, a recent study comparing COVID-19 vaccine hesitancy among U.S. military veterans and non-veterans in Utah found notable differences: the veterans were more likely to be either vaccine-accepting or strongly opposed, whereas the non-veterans included more individuals who were uncertain [30].

Our data-driven approach used patterns of co-occurring responses to identify distinct classes of vaccine hesitancy concerns (e.g., vaccine safety, trust in government and institutions). Many of the individual survey responses about safety and trust align with vaccine hesitancy determinants identified by the SAGE Working Group under contextual influences and individual and group influences, underscoring the importance of considering these domains when interpreting patterns of hesitancy.

An examination of the differences between February 2021, when the survey largely measured prospective hesitancy and May 2021, the point of universal vaccine eligibility, provides additional insights. Overall, the relative size of the hesitant population decreased, from 19.1% in February to 13.3% in May, in line with previous research (e.g., [15]). However, in May, 22% of unvaccinated respondents indicated that they definitely or probably would take a vaccine if it were offered to them that day, suggesting that access to the vaccine in May 2021 remained an issue.

Similar class types emerged across the two time points; however, their relative sizes in the sample changed. From February to May, the proportion in the ambiguous class decreased slightly and the relative size of the distrustful class increased from 22.1% to 27.6%, displacing health-concerned hesitancy as the second largest class. The decrease in the prevalence of hesitant respondents suggests that formerly hesitant respondents became vaccine accepting by May. This analysis cannot determine from which hesitant class those defections occurred, as the study did not track individual respondents over time. However, if hesitancy type persisted over the course of the rollout (that is, if the reason someone was hesitant in February did not change in May) and the primary change was conversion from vaccine hesitant to vaccine accepting, the change in relative class size would indicate indicate that the distrustful class was less receptive to efforts to increase vaccine acceptance.

Importantly, when we examined subgroups of White, Black, and Hispanic respondents, the flip of distrustful and hesitant class sizes was only seen for Whites. For Black and Hispanic participants, the health-concerned class was still larger than the distrustful class. If individuals who expressed health concerns in February were more open to being converted to vaccine accepting by May, these results support concerns about differential access to the vaccine during the period of limited supply. Previous research has shown that COVID-19 vaccine uptake between December 2020 and January 2021 varied by racial and ethnic groups [31] and was mediated by factors that varied between racial and ethnic groups, including employment, household income, marital status, and chronic health conditions, which contributed to lower rates of vaccination among Black and Hispanic populations compared to Whites [32,33].

Also, distrust of the health care system among Black and Hispanic individuals in the US has been studied in general and in the context of COVID-19 vaccines [34], and vaccine hesitancy was expected among these populations. However, White hesitancy initially appeared lower [35] and was not as well studied. This study identified a potentially important distinction. At both time points, in the distrustful class for Black and Hispanic groups, health-related reasons had a higher probability of selection than in the White distrustful group. For example, “distrustful” Black and Hispanic respondents were more likely to have concerns about allergic reactions than an individual in the White distrustful class (16 and 11 percentage points, respectively) (see Tables C and D in S1 Appendix). This suggests that for Black or Hispanic respondents, distrust of the vaccine or government was rooted in different concerns than the kind of distrust of the government that was prevalent among Whites in February and May, 2021, when the pandemic had been heavily politicized in the US. Research has shown that people living in counties with higher support from Trump in the 2020 presidential election were more likely to be vaccine hesitant [31], and those who identified as Democrats showed higher rates of vaccine acceptance than those who identified as Republican or Independent [28].

This ideological divide is not unique to the U.S.; across 21 European countries, far-right voters were significantly more likely to be COVID-19 vaccine hesitant than centrist voters [36]

However, context matters. While vaccine hesitancy is higher among conservatives than liberals in the U.S., some predictors of vaccination behavior cut across party lines. Pew Research data show that prior-year flu vaccination strongly predicts COVID-19 vaccine acceptance among both liberals and conservatives, while exposure to misinformation increases hesitancy among conservatives and moderates but not liberals [37].

Our study adds to the literature by synthesizing self-reported reasons for vaccine hesitancy and demonstrating Latent Class Analysis as a data-driven framework for identifying nuanced subgroup patterns. Because of pre-existing disparities in healthcare access and outcomes, this analysis focuses on racial and ethnic variation, which was a heightened concern during the vaccine rollout. Future research could extend these analyses to other demographic factors linked to hesitancy, including education, occupation, prior flu vaccination, or county-level political affiliation [15,19,38].

Other studies underscore the role of trust in government in shaping public health behaviors, including mask use and social distancing [39]. Trust emerged here as a persistent driver of hesitancy, underscoring the challenge of rebuilding institutional trust.

Still, our analyses also suggest that change is possible. During late 2020 and 2021, outreach campaigns were initiated by many individuals and groups within their own communities, such as efforts by Black doctors and nurses [40]. Health organizations, including the CDC, provided resources and toolkits to promote vaccine uptake among non-White race and ethnic groups [41].

This study focused on the period of the initial COVID-19 vaccine rollout. However, the consistent emergency of the different hesitancy types suggests the identification of constructs with continued salience, as researchers continue to identify sources of continuing and increasing vaccine hesitancy [42].

## Limitations and strengths

As the models used for this analysis do not support survey weights, the analysis is based on an unweighted sample recruited from the Facebook Monthly Active User Base, which is more female, White, and college educated than the US adult population. It was also not possible to link respondents across time to determine if respondents were included in both time periods. A sensitivity analysis to see if the relative size of classes would have been likely to changed if survey weights suggested such a change would be unlikely. Due to the scale of the survey, no fill-in option was available for the respondents to provide their own reasons for hesitancy, as the scale of the survey prohibited coding of write-in data, so responses were limited to the list of options provided on the survey. Additionally, this study relied on a common practice to categorize a Likert scale question on vaccine hesitancy into hesitant/not hesitant. Given the different hesitancy classes identified in this study, future work should examine whether those who say they “probably would not” or “definitely would not” get the vaccine show different hesitancy classes, or as seen in this study, different relative sizes of similar hesitancy classes.

A strength of the analysis is that the scale of the survey enabled important subgroup analyses, even when restricting the sample to only hesitant responses. Latent class analysis is only feasible for reasonably large data sets, which were available for this study.

Future work could take advantage of this scale model to hesitancy types for different groupings; for example, those who had or had not recieved a prior-year flu vaccine. Additionally, the ability to compare responses from February and May enables insight into changes that occurred over the rollout, including responses public health campaigns, increased eligibility, and the possibility for individuals to see others receive a vaccine.

## Conclusion

COVID-19 vaccine hesitancy is a heterogeneous construct, and officials need to consider multiple, distinct types of hesitancy related to health concerns and government distrust. Further, in the US, types and their prevalence vary among racial and ethnic subgroups.

## Supporting information

S1 Appendix Methods supplement and Survey questions and response probabilities. (PDF)
