# Supplementary material for: Health concerns and government distrust: Variation in types of COVID-19 vaccine hesitancy by racial and ethnic group before and at universal vaccine eligibility in the US
Source: PLOS Glob Public Health. 2026 Jun 3;6(6):e0004942. doi: 10.1371/journal.pgph.0004942 (PMC13232814; doi:10.1371/journal.pgph.0004942)
Supplement: S1 Appendix — (PDF) [file pgph.0004942.s001.pdf]

S1 Appendix

Methods Supplement

The model used for the Latent Class Analysis is as follows.

- We have J categorical manifest variables that can take on values in $\{0, 1\}$. In our case, these are the hesitancy reasons, which can either be selected or unselected.
- There are G groups, each of size n_g, where $\sum_{g=1}^{G} n_g = N$, the total number of individuals. Each individual is observed to fall into one group. In this analysis, the groups are race/ethnicity groups. When fit across all race/ethnicity groups, $G = 1$; models with $G = 3$ were used to separately model White, Black, and Hispanic respondents.
- Y_{gij}, the categorical manifest variable, represents the response of individual i in group g on variable j, and it is either 1 if that reason was selected or 0 if it was not.
- There are then R latent classes assumed to exist within each of the G groups. Membership of individuals in latent classes is not observed, but must be inferred from the data.

We say that the probability of selecting a variable j in class r and group g is π_{grj}. The π_{gr} vectors thus define the latent class: each class has a different pattern of responses to the J manifest variables.

The mixing proportions for class r and group g (the probability that a respondent in group g falls into latent class r) are represented with p_{gr}, such that $\sum_{r=1}^{R} p_{gr} = 1$ for each group g.

The probability of observing a specific set of responses Y_{gi} for individual i in group g and class r (i.e., across the J variables) is

$$f(Y_{gi}; \pi_{gr}) = \prod_{j=1}^{J} (\pi_{grj})^{Y_{gij}}.$$

Then, the probability density function across all classes in group g is

$$\Pr(Y_{gi} \mid \pi_g, p_g) = \sum_{r=1}^{R} p_{gr} \prod_{j=1}^{J} (\pi_{grj})^{Y_{gij}}.$$

When fitting the model, we estimate the parameters for each group and latent class: $\hat{p}_{gr}, \hat{\pi}_{grj}$. (Further detail on fitting the LCA model can be found in [1] and [2].) The $\hat{\pi}_{grj}$ estimates can be plotted to show the estimated response patterns within each group and latent class. The mixing

proportions $\hat{p}_{gr}$ give the average membership probability of each class, i.e., the estimated proportion of respondents in group g who are in latent class r.

Let r_i be the unobserved latent class membership for individual i, so $r_i = r$ if respondent i is in latent class r. We can then estimate the posterior probability of each individual i falling into a class r given its group g and its observed responses using Bayes' rule:

$$\widehat{\Pr}(r_i = r \mid Y_{gi}) = \frac{\hat{p}_{gr} f(Y_{gi}; \hat{\pi}_{gr})}{\sum_{q=1}^{R} \hat{p}_{gq} f(Y_{gi}; \hat{\pi}_{gq})}.$$

As the latent class model does not incorporate survey weights, we conducted a sensitivity analysis of the overall model (with $G = 1$). Let w_i be the survey weight for individual i. These weights are scaled so the sum of the weights each day is the US adult population; thus w_i can be seen as representing how many US adults (on one day) are represented by this individual's survey response. (For details on the survey weights, see the User Guide for the COVID-19 Trends and Impact Survey Weights, available at [3].) After trimming outlying high weights,

$$\frac{\sum_{i=1}^{N} w_i \widehat{\Pr}(r_i = r \mid Y_{gi})}{\sum_{i=1}^{N} \widehat{\Pr}(r_i = r \mid Y_{gi})}$$

is the weighted average survey weight for latent class r. On the overall model with three latent classes, the three means were 9384, 9545, and 9151; kernel density estimates of the weight distributions were visually almost identical. This demonstrates that the weights do not dramatically differ between latent classes. Large differences would indicate differences in weight between classes, suggesting that weighting the data would significantly change the proportion of respondents in each latent class.

CTIS Questions Used in This Analysis

Initial Vaccination Questions

The first vaccine question was asked of all respondents. It is item V1 in the survey data.

Have you had a COVID-19 vaccination?

- Yes
- No
- I don't know

All respondents who did not say "yes" to the first question were then asked the following question, which corresponds to item V3.

If a vaccine to prevent COVID-19 were offered to you today, would you choose to get vaccinated?

- Yes, definitely
- Yes, probably
- No, probably not
- No, definitely not

Vaccine Hesitancy Reasons

This question was offered to respondents who indicated that they "probably would", "probably would not", or "definitely would not" accept a COVID-19 vaccine. It corresponds to items V5a-c.

Which of the following, if any, are reasons that you only probably *(or "probably wouldn't" or "definitely wouldn't", as applicable)* would choose to get a COVID-19 vaccine? Please select all that apply.

- I am concerned about possible side effects of a COVID-19 vaccine.
- I am concerned about having an allergic reaction to a COVID-19 vaccine.
- I don't know if a COVID-19 vaccine will work.
- I don't believe I need a COVID-19 vaccine.
- I don't like vaccines generally.
- My doctor has not recommended it.
- I plan to wait and see if it is safe and may get it later.
- I think other people need it more than I do right now.
- I am concerned about the cost of a COVID-19 vaccine.
- I don't trust the government.
- It is against my religious beliefs.
- I don't trust COVID-19 vaccines.
- I have a health condition and am concerned about the safety of the vaccine for people with my condition.
- I am currently/planning to be pregnant and/or breastfeeding and do not want to get vaccinated at this time.
- Other

Race and Ethnicity Questions

Both of the following questions were asked of all respondents, regardless of their answer to the first question, and multiple options could be selected for the second question. Responses were then recoded to one of eight racial/ethnic groups. These questions correspond to items D6 and D7.

Are you of Hispanic, Latino, or Spanish origin?

- Yes
- No, not of Hispanic, Latino, or Spanish origin

What is your race?

- American Indian or Alaska Native
- Asian
- Black or African American
- Native Hawaiian or other Pacific Islander
- White
- Some other race

Response Probability Estimates with Standard Errors

Reason	Ambiguous	Health-concerned	Distrustful
Side effects	0.1499 (0.000003701)	0.8978 (0.000009712)	0.8262 (0.000006959)
Allergic reaction	0.06893 (0.000001252)	0.5015 (0.00001215)	0.46 (0.00001098)
Don't know if it works	0.04448 (0.0000008209)	0.3335 (0.000009211)	0.5645 (0.00001074)
Don't need	0.1578 (0.000001938)	0.1122 (0.000005159)	0.6095 (0.00001159)
Don't like vaccines	0.06227 (0.0000008241)	0.06654 (0.000002717)	0.3523 (0.000009116)
Not yet recommended by Dr	0.04066 (0.0000005558)	0.1035 (0.000003479)	0.1647 (0.000005393)
Wait and see	0.2581 (0.000003651)	0.7474 (0.00001113)	0.3696 (0.00001121)
Others need it more	0.1597 (0.000002014)	0.3699 (0.000009575)	0.2388 (0.000007936)
Cost	0.007836 (0.000000132)	0.07395 (0.0000024)	0.09783 (0.000003452)
Don't trust vaccines	0.1946 (0.000002674)	0.2986 (0.00001108)	0.9481 (0.000004048)
Don't trust government	0.1657 (0.000002141)	0.1811 (0.000008234)	0.8093 (0.000008808)
Religious beliefs	0.03213 (0.0000004119)	0.007475 (0.0000004059)	0.1511 (0.000004838)
Health condition	0.07025 (0.000001004)	0.2219 (0.000006839)	0.1673 (0.000005869)
Pregnant/breastfeeding	0.0483 (0.0000006318)	0.06473 (0.000002244)	0.07054 (0.000002488)
Other	0.1517 (0.000001859)	0.02666 (0.000001245)	0.1266 (0.000003956)

Table A: Response probability estimates ($\hat{\pi}$) for three latent classes in February 2021, with standard errors.

Reason	Ambiguous	Health-concerned	Distrustful
Side effects	0.1312 (0.000009399)	0.845 (0.00001074)	0.8751 (0.00002696)
Allergic reaction	0.04693 (0.000002467)	0.4463 (0.0000177)	0.4809 (0.00002949)
Don't know if it works	0.02971 (0.000001423)	0.5022 (0.00001734)	0.254 (0.00001817)
Don't need	0.2371 (0.000006633)	0.7146 (0.00001715)	0.1727 (0.00001733)
Don't like vaccines	0.05733 (0.000001913)	0.3347 (0.00001434)	0.06703 (0.00000624)
Not yet recommended by Dr	0.04267 (0.000001427)	0.178 (0.000009252)	0.1003 (0.000008012)
Wait and see	0.1952 (0.000007266)	0.3229 (0.00001665)	0.6648 (0.00002991)
Others need it more	0.059 (0.000001995)	0.1433 (0.000008525)	0.1748 (0.000013)
Cost	0.003215 (0.0000001368)	0.06545 (0.000003898)	0.02925 (0.000002329)
Don't trust vaccines	0.2544 (0.00000782)	0.963 (0.000004638)	0.3544 (0.00002744)
Don't trust government	0.2297 (0.000006673)	0.8496 (0.00001221)	0.2021 (0.00002224)
Religious beliefs	0.04377 (0.000001379)	0.189 (0.000009754)	0.01346 (0.000001578)
Health condition	0.0704 (0.000002579)	0.1871 (0.00001048)	0.2616 (0.0000185)
Pregnant/breastfeeding	0.04433 (0.000001445)	0.07008 (0.000004193)	0.07148 (0.000005639)
Other	0.2125 (0.000006051)	0.1652 (0.00000816)	0.04378 (0.000004661)

Table B: Response probability estimates ($\hat{\pi}$) for three latent classes in May 2021, with standard errors.

**White**			**February**
Reason	Ambiguous	Health-concerned	Distrustful
Side effects	0.1457 (0.0027)	0.8939 (0.0041)	0.8089 (0.0035)
Allergic reaction	0.0657 (0.0015)	0.4651 (0.0045)	0.4063 (0.0041)
Don't know if it works	0.0386 (0.0012)	0.3331 (0.004)	0.5322 (0.0041)
Don't need	0.1767 (0.002)	0.1289 (0.0031)	0.6144 (0.0043)
Don't like vaccines	0.0561 (0.0012)	0.0689 (0.0021)	0.3207 (0.0037)
Not yet recommended by Dr	0.0404 (0.001)	0.1115 (0.0025)	0.1402 (0.0027)
Wait and see	0.2705 (0.0027)	0.7577 (0.0043)	0.333 (0.0042)
Others need it more	0.1763 (0.002)	0.4097 (0.0042)	0.207 (0.0034)
Cost	0.0061 (0.0005)	0.0721 (0.002)	0.0729 (0.002)
Don't trust vaccines	0.1797 (0.0022)	0.288 (0.0043)	0.9516 (0.0026)
Don't trust government	0.1572 (0.002)	0.1787 (0.0037)	0.8059 (0.0038)
Religious beliefs	0.0236 (0.0008)	0.0063 (0.0007)	0.1156 (0.0024)
Health condition	0.0759 (0.0014)	0.2213 (0.0034)	0.1412 (0.0028)
Pregnant/breastfeeding	0.0517 (0.0011)	0.0646 (0.002)	0.049 (0.0017)
Other	0.1513 (0.0019)	0.028 (0.0015)	0.1167 (0.0024)

**Black**			
Reason	Ambiguous	Health-concerned	Distrustful
Side effects	0.1998 (0.0079)	0.9404 (0.0099)	0.872 (0.0086)
Allergic reaction	0.0958 (0.0049)	0.6301 (0.0136)	0.5635 (0.0122)
Don't know if it works	0.0479 (0.0033)	0.3093 (0.0111)	0.5589 (0.0124)
Don't need	0.0831 (0.0038)	0.0569 (0.0066)	0.4052 (0.0124)
Don't like vaccines	0.0664 (0.0034)	0.0562 (0.0062)	0.3486 (0.0115)
Not yet recommended by Dr	0.0447 (0.0028)	0.1105 (0.007)	0.1435 (0.0082)
Wait and see	0.3325 (0.0075)	0.7495 (0.0117)	0.5073 (0.0125)
Others need it more	0.1032 (0.0043)	0.2364 (0.0098)	0.2478 (0.0102)
Cost	0.0081 (0.0013)	0.0564 (0.0051)	0.0877 (0.0064)
Don't trust vaccines	0.2208 (0.0062)	0.3309 (0.0133)	0.9222 (0.0094)
Don't trust government	0.1363 (0.0049)	0.1906 (0.0113)	0.7521 (0.0128)
Religious beliefs	0.0383 (0.0025)	0.0075 (0.0026)	0.1138 (0.0071)
Health condition	0.0908 (0.0042)	0.3146 (0.0109)	0.1933 (0.0097)
Pregnant/breastfeeding	0.0449 (0.0028)	0.0445 (0.0049)	0.0863 (0.0063)
Other	0.1079 (0.0043)	0.0276 (0.0039)	0.0602 (0.0053)

**Hispanic**			
Reason	Ambiguous	Health-concerned	Distrustful
Side effects	0.1929 (0.0058)	0.9221 (0.0082)	0.8419 (0.0094)
Allergic reaction	0.0914 (0.0037)	0.5904 (0.0108)	0.5447 (0.0127)
Don't know if it works	0.0622 (0.003)	0.4137 (0.0098)	0.6548 (0.012)
Don't need	0.1181 (0.0035)	0.0912 (0.0064)	0.6155 (0.0131)
Don't like vaccines	0.0667 (0.0027)	0.0648 (0.005)	0.4007 (0.0118)
Not yet recommended by Dr	0.0365 (0.002)	0.0735 (0.0049)	0.1987 (0.0097)
Wait and see	0.2874 (0.0057)	0.7683 (0.0097)	0.4495 (0.013)
Others need it more	0.1926 (0.0044)	0.3605 (0.0092)	0.3393 (0.0121)
Cost	0.0104 (0.0012)	0.0975 (0.0054)	0.1721 (0.0093)
Don't trust vaccines	0.1761 (0.0045)	0.3433 (0.0099)	0.9415 (0.0072)
Don't trust government	0.1381 (0.0039)	0.1839 (0.0087)	0.8204 (0.0111)
Religious beliefs	0.0365 (0.002)	0.0079 (0.0021)	0.2195 (0.0099)
Health condition	0.0633 (0.0028)	0.1858 (0.0073)	0.2077 (0.0102)
Pregnant/breastfeeding	0.06 (0.0026)	0.0775 (0.005)	0.1273 (0.0079)
Other	0.1124 (0.0034)	0.0204 (0.003)	0.1405 (0.0078)

Table C: Response probability estimates ($\hat{\pi}$) for three latent classes by race/ethnicity in February 2021, with standard errors.

**White**			**May**
Reason	Ambiguous	Health-concerned	Distrustful
Side effects	0.1314 (0.0042)	0.8615 (0.007)	0.8398 (0.0041)
Allergic reaction	0.0425 (0.0021)	0.4562 (0.007)	0.4085 (0.005)
Don't know if it works	0.0253 (0.0015)	0.2417 (0.0055)	0.4708 (0.005)
Don't need	0.2687 (0.0037)	0.1845 (0.0055)	0.6961 (0.005)
Don't like vaccines	0.0548 (0.0018)	0.058 (0.003)	0.2894 (0.0043)
Not yet recommended by Dr	0.0417 (0.0016)	0.111 (0.0038)	0.1486 (0.0034)
Wait and see	0.1961 (0.0037)	0.6665 (0.0072)	0.3072 (0.0048)
Others need it more	0.0565 (0.0019)	0.1839 (0.0048)	0.1144 (0.0031)
Cost	0.002 (0.0004)	0.0266 (0.0019)	0.0401 (0.0018)
Don't trust vaccines	0.2566 (0.0039)	0.3203 (0.0069)	0.9618 (0.0028)
Don't trust government	0.2304 (0.0035)	0.1766 (0.0059)	0.8386 (0.0044)
Religious beliefs	0.0353 (0.0014)	0.0109 (0.0015)	0.141 (0.0033)
Health condition	0.0726 (0.0022)	0.2663 (0.0057)	0.1628 (0.0036)
Pregnant/breastfeeding	0.044 (0.0016)	0.0745 (0.0031)	0.0495 (0.0021)
Other	0.2062 (0.0033)	0.0438 (0.0028)	0.139 (0.0032)

**Black**			
Reason	Ambiguous	Health-concerned	Distrustful
Side effects	0.1862 (0.0168)	0.9146 (0.0198)	0.8544 (0.0178)
Allergic reaction	0.0918 (0.0104)	0.6388 (0.0271)	0.5691 (0.0245)
Don't know if it works	0.0464 (0.0065)	0.2387 (0.0192)	0.5708 (0.0242)
Don't need	0.1127 (0.009)	0.0877 (0.0145)	0.5311 (0.0255)
Don't like vaccines	0.0525 (0.0065)	0.1018 (0.0139)	0.3965 (0.0229)
Not yet recommended by Dr	0.0453 (0.0057)	0.0736 (0.0113)	0.1472 (0.0158)
Wait and see	0.2656 (0.0145)	0.6686 (0.0232)	0.4781 (0.0241)
Others need it more	0.0562 (0.0065)	0.0991 (0.0133)	0.2016 (0.0179)
Cost	0.0073 (0.0025)	0.0447 (0.0086)	0.0652 (0.0108)
Don't trust vaccines	0.2554 (0.0136)	0.3859 (0.0246)	0.9652 (0.0133)
Don't trust government	0.1661 (0.011)	0.2056 (0.0213)	0.7934 (0.0223)
Religious beliefs	0.0592 (0.0065)	0.0193 (0.0065)	0.1778 (0.0168)
Health condition	0.09 (0.009)	0.3629 (0.0217)	0.2323 (0.0202)
Pregnant/breastfeeding	0.0546 (0.0063)	0.0452 (0.0094)	0.0872 (0.0123)
Other	0.1547 (0.0103)	0.0363 (0.0092)	0.1181 (0.014)

**Hispanic**			
Reason	Ambiguous	Health-concerned	Distrustful
Side effects	0.1526 (0.0101)	0.9284 (0.0133)	0.8416 (0.0137)
Allergic reaction	0.0677 (0.0057)	0.549 (0.0165)	0.5168 (0.0191)
Don't know if it works	0.0441 (0.0046)	0.3364 (0.0144)	0.5792 (0.0181)
Don't need	0.1893 (0.0077)	0.1478 (0.0121)	0.7801 (0.0169)
Don't like vaccines	0.0675 (0.0049)	0.0896 (0.0087)	0.4353 (0.0177)
Not yet recommended by Dr	0.0422 (0.0039)	0.0675 (0.0075)	0.2738 (0.0161)
Wait and see	0.2413 (0.0094)	0.7229 (0.0154)	0.382 (0.0187)
Others need it more	0.095 (0.0057)	0.1817 (0.0113)	0.2338 (0.0161)
Cost	0.0068 (0.0016)	0.0359 (0.0053)	0.1411 (0.0136)
Don't trust vaccines	0.2373 (0.0089)	0.4459 (0.0155)	0.9616 (0.0078)
Don't trust government	0.192 (0.008)	0.2413 (0.0143)	0.8865 (0.0136)
Religious beliefs	0.0541 (0.0043)	0.0141 (0.0041)	0.3563 (0.0172)
Health condition	0.0695 (0.0051)	0.2129 (0.0119)	0.2436 (0.0162)
Pregnant/breastfeeding	0.0637 (0.0047)	0.092 (0.0084)	0.1583 (0.0135)
Other	0.181 (0.0075)	0.0299 (0.0057)	0.2003 (0.0131)

Table D: Response probability estimates ($\hat{\pi}$) for three latent classes by race/ethnicity in May 2021, with standard errors.

References

1. Linzer DA, Lewis JB. poLCA: An R Package for Polytomous Variable Latent Class Analysis. Journal of Statistical Software. 2011;42(10):1–29. doi:10.18637/jss.v042.i10.

2. Kim Y, Chung H. Introduction to glca package; 2021. https://cran.r-project.org/web/packages/glca/vignettes/glca.html.

3. Reinhart A, Mejia R, Tibshirani RJ. COVID-19 Trends and Impact Survey (CTIS), United States, 2020-2022; 2025. Inter-university Consortium for Political and Social Research. Available from: `https://doi.org/10.3886/ICPSR39207.v1`.
